# Social distancing causally impacts the spread of SARS-CoV-2: a U.S. nationwide event study

**DOI:** 10.1186/s12879-022-07763-y

**Published:** 2022-10-13

**Authors:** Louis Gagnon, Stephanie Gagnon, Jessica Lloyd

**Affiliations:** 1 Smith School of Business, Queen’s University, Kingston, ON K7L 3N6 Canada; 2 School of Medicine, Royal College of Surgeons in Ireland, Dublin, Republic of Ireland

**Keywords:** SARS-CoV-2, Covid-19, Social distancing, Social mobility, Staggered difference-in-difference, Propensity score matching, George Floyd protests

## Abstract

We assess the causal impact of social distancing on the spread of SARS-CoV-2 in the U.S. using the quasi-natural experimental setting created by the spontaneous relaxation of social distancing behavior brought on by the protests that erupted across the nation following George Floyd’s tragic death on May 25, 2020. Using a difference-in-difference specification and a balanced sample covering the [− 30, 30] day event window centered on the onset of protests, we document an increase of 1.34 cases per day, per 100,000 population, in the SARS-CoV-2 incidence rate in protest counties, relative to their propensity score matching non-protest counterparts. This represents a 26.8% increase in the incidence rate relative to the week preceding the protests. We find that the treatment effect only manifests itself after the onset of the protests and our placebo tests rule out the possibility that our findings are attributable to chance. Our research informs policy makers and provides insights regarding the usefulness of social distancing as an intervention to minimize the spread of SARS-CoV-2.

## Introduction

The highly contagious novel coronavirus, severe acute respiratory syndrome coronavirus-2 (SARS-CoV-2), responsible for coronavirus disease 2019 (COVID-19), emerged in December 2019 in Wuhan city, Hubei province, China [1]. The initial COVID-19 outbreak quickly evolved into a pandemic [2], and as of June 2020, SARS-CoV-2 has reached over 180 countries and regions, with the total number of confirmed cases surpassing 10 million globally [3]. COVID-19 has spread throughout the United States (U.S.) at an unparalleled rate, infecting over 2.5 million individuals and claiming over 125,000 lives [4]. Global public health measures aimed at reducing the spread of SARS-CoV-2 have been designed in consideration of the virus’s specific transmission properties [5]. SARS-CoV-2 can be transmitted through various modes, including person-to-person contact and the spread of respiratory droplets, which can travel across a minimum distance of 6 feet (2 m) [6, 7]. Numerous countries have introduced social distancing, defined as the maintenance of at least a 6 foot interpersonal physical separation, to minimize direct transmission from infected individuals [8].

In the U.S., individual states have been granted the authority to design their own COVID-19 mitigation strategy, therefore, the extent and type of social distancing policies adopted differs across states [9]. Research examining state-imposed restrictions has found a reduction in the doubling rate of SARS-CoV-2 among U.S. states [10], as well as the daily growth rate of COVID-19 cases across U.S counties after the imposition of social distancing measures [11, 12]. Other research has suggested that rather than reducing the daily growth rate of COVID-19, social distancing merely stabilizes the spread of SARS-CoV-2 in the U.S [10]. Additionally, when examining the effectiveness of social distancing, studies have used social mobility as a measure of social distancing [11, 13–15]. However, mobility represents an imperfect proxy for social distancing because individuals can be mobile while still maintaining the required minimum 6 foot separation from others to prevent viral transmission. Furthermore, although evidence suggests there is an association between social distancing and the spread of SARS-CoV-2, the causal impact of social distancing on the spread of SARS-Cov-2 is still unknown.

In this study, we examine the causal impact of a spontaneous relaxation of social distancing measures on the spread of SARS-CoV-2. The nationwide mass protests precipitated by George Floyd’s tragic death on May 25, 2020 prompted an abrupt relaxation of social distancing behavior across the U.S [16]. The unpredictable nature of the protests created a natural experimental setting to assess for causality. In this study, instead of using mobility as a proxy for social distancing, we control for the increase in mobility during the protest period in order to hone in on the direct effect of social distancing. We also explicitly control for the concurrent relaxation of state-imposed restrictions to account for variations in social distancing restrictions across states.

## Methods

This study uses publicly accessible data exclusively and all statistical methods employed herein comply with relevant guidelines and regulations.

### Data and sample description

We source our U.S. COVID-19 data from the John Hopkins Whiting School of Engineering’s Center for Systems Science and Engineering’s GitHub repository [17]. This data consists of confirmed cases in each county at the end of every day since the start of the outbreak in late January 2020. We calculate the number of new cases for each county and each day by subtracting the cumulative number of confirmed cases at the end of the day from the number of cumulative cases from the previous day.

We obtain our county-level population data and our county-level demographic data from the U.S. Census Bureau [18]. We extract our county-level Gross Domestic Product (GDP) data from the U.S. Bureau of Economic Analysis’ (BEA) Regional Economic Accounts database (Table CAGDP1) [19]. We retrieve county-level data on the prevalence of obesity, diabetes, smoking, and hypertension from the University of Washington’s Institute for Health Metrics and Evaluation (IHME) [20]. The hypertension and obesity data are for the years 2009 and 2011, respectively, and the diabetes and smoking prevalence data are for 2012. The IHME reports hypertension and obesity data for females and males separately, so we construct a population-weighted average measure for these two covariates based on the proportion of females and males in each county, as reported by the U.S. Census Bureau.

The social distancing restrictions data is from the University of Washington’s State-Level Social Distancing Policies in Response to the 2019 Novel Coronavirus in the U.S. repository [21]. The social distancing restrictions include: (1) restrictions on public gatherings exceeding 5, 10, 25, 50, 100, 250, 500, or 1000 people, (2) limits on restaurant operations, (3) closure of specific businesses, e.g. fitness centres, gyms, casinos, etc., (4) closure of non-essential businesses, (5) stay-at-home orders for non-essential activities, (6) state curfews on non-essential activities, (7) mandated quarantines for people entering the state, (8) travel restrictions prohibiting residents from leaving the state, non-residents from entering the state, or residents from travelling across counties within the state, (9) self-isolation requirement for individuals with confirmed COVID-19 incidence, and (10) mandatory wearing of masks or other mouth and nose coverings in public places. We construct our social distancing restrictions index by adding the number of restrictions that are in place in a state on any given day, based on the date at which each restriction is enacted, relaxed, or expired.

We obtain our mobility data from the Descartes Labs [22]. This data consists of mobility indexes calculated at the end of every day and aggregated at the county level. The indexes, which we will refer to as the social mobility indexes, are based on geolocation reports from smartphones and other mobile devices, and track the movements of individual mobile phone subscribers. The methodology employed to construct these indexes is described in Warren et al. [23]. The mobility index data is available at a daily frequency from March 1, 2020, for 2669 counties.

Finally, we construct a comprehensive list of protests that took place across the U.S. based on the List of George Floyd protests in the United States assembled by Wikipedia [24]. At the time of writing, the main Wikipedia page cited 134 news articles from national, regional, and local media outlets, and the secondary pages cited hundreds more. From these media citations, we extracted the location and the date at which the protests reportedly took place, as well as the estimated number of individuals involved in each protest. We complement this process with a search on the Dow Jones Factiva database [25]. The onset of the protests among the counties in which protests took place, i.e. the treatment, is staggered across time and ranges between May 26, 2020, and June 7, 2020, so we center our experiment on the first protest date in each treated county, as opposed to the date of George Floyd’s death, May 24, 2020. Therefore, the George Floyd protests produce a quasi-natural experimental setting with staggered treatment dates, rather than a single treatment date setting.

Our sample period begins on March 1, 2020, when the social mobility data becomes available and ends on July 7, 2020. This ending date enables us to carry out our estimation on a balanced panel dataset consisting of a 30-day event window centered on the onset of the protests in each protest county. Our sampling procedure yields a panel dataset consisting of a total of 256,202 county-days representing 2617 (541 protest and 2076 non-protest) counties from all fifty states with incidence rate and covariates data available for our entire estimation window. From this dataset, we form covariate-balanced treatment and control groups using the propensity score matching technique described below and carry out our estimation of the treatment effect.

**Fig. 1:**
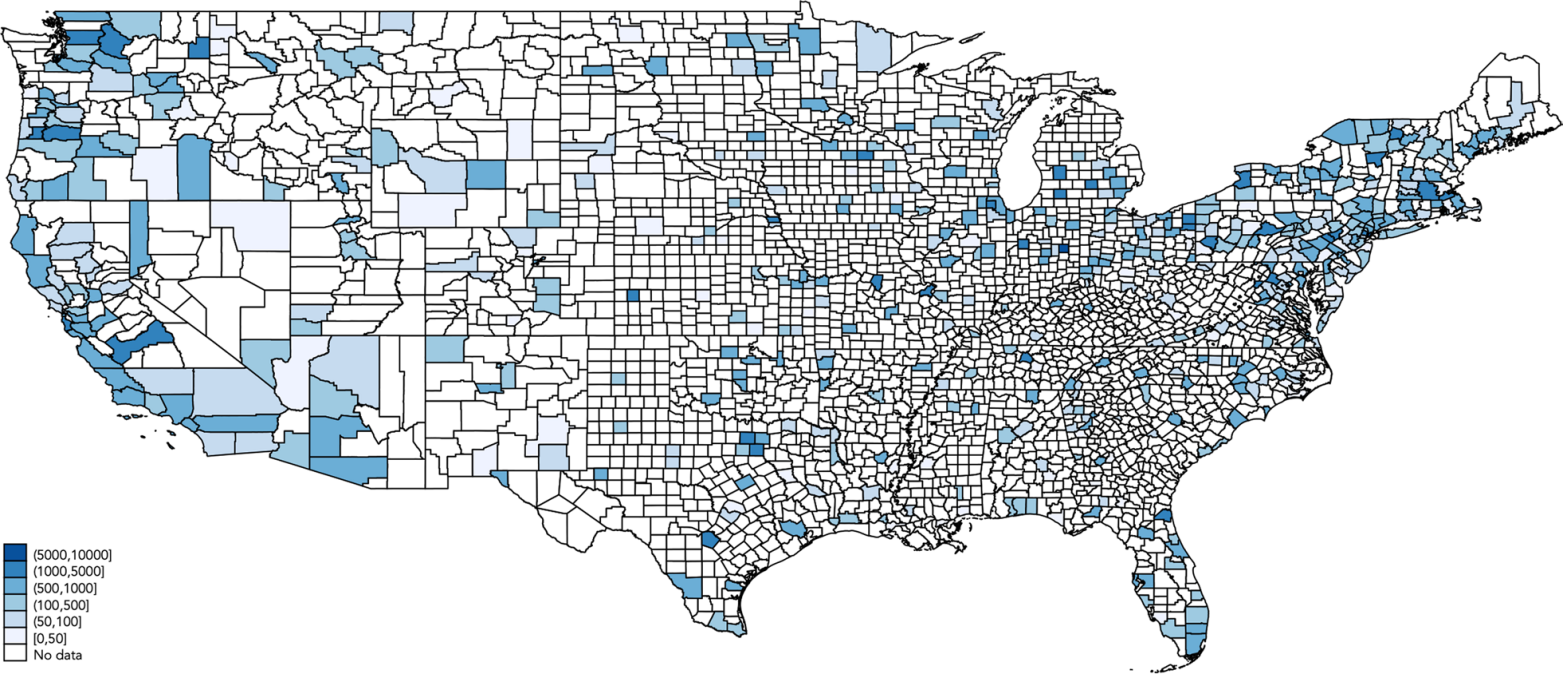
Counties involved in protests. This figure identifies the counties in which protests took place, according to media reports, along with the number of participants involved in the first protest that took place within each county. Counties within the states of Alaska and Hawaii are not shown, but they are included in our sample

**Fig. 2:**
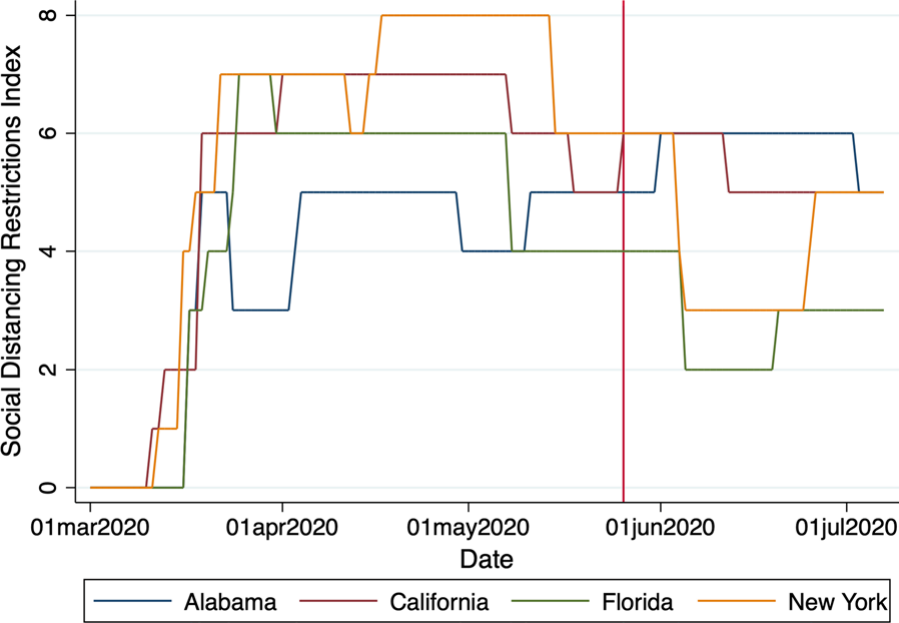
Social distancing restrictions index. This figure shows the evolution of our social distancing restrictions index from March 1, 2020, to July 7, 2020, for the states of Alabama, California, Florida, and New York. The vertical line corresponds to May 26, 2020, the day of the protests’ onset

**Fig. 3:**
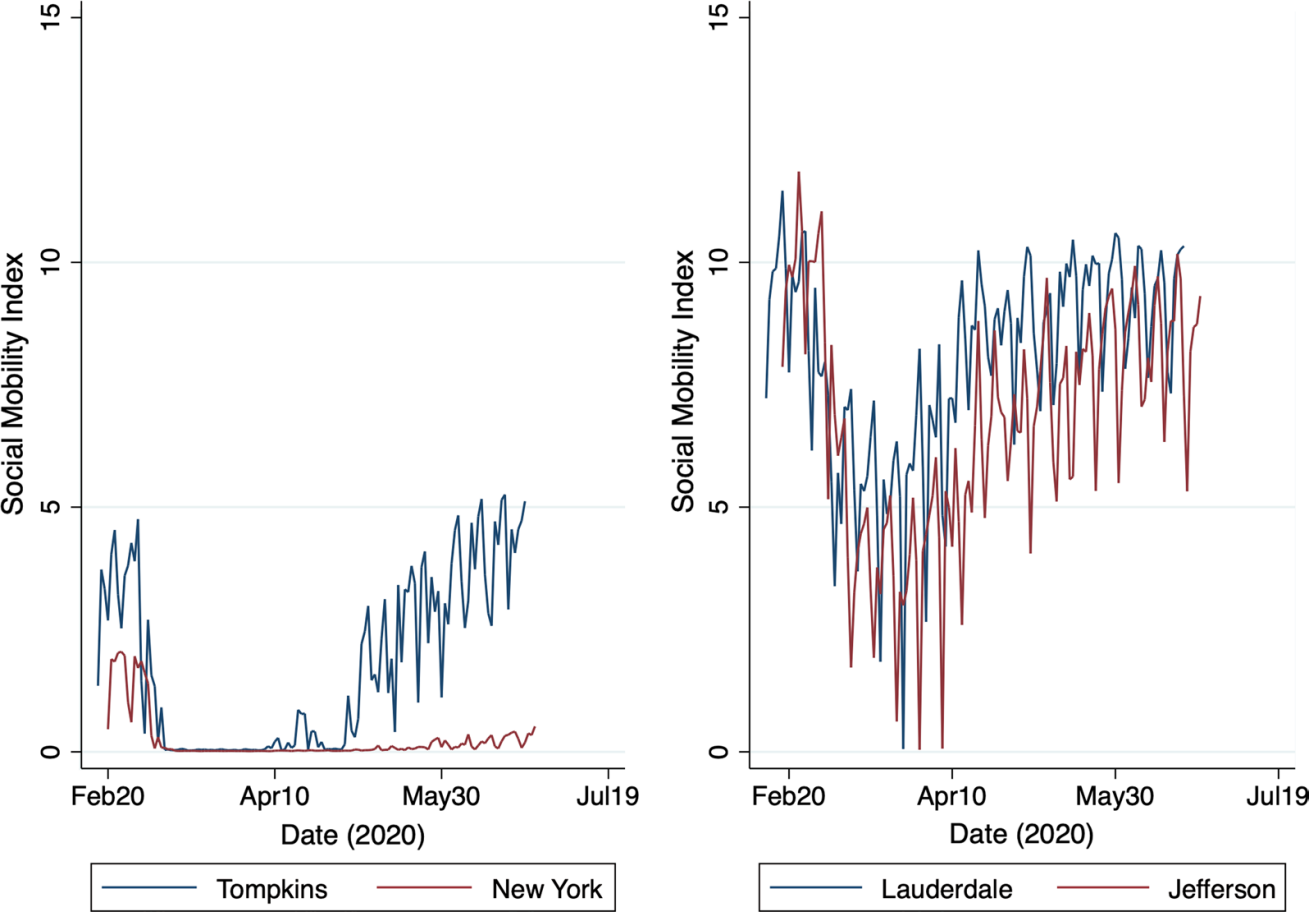
Social mobility index. This figure shows the evolution of the social mobility index from March 1, 2020, to July 7, 2020, for Tompkins and New York counties in the state of New York, and for Lauderdale and Jefferson counties in the state of Alabama

We report descriptive statistics for new and cumulative SARS-CoV-2 cases in Table 1, broken down by state, along with the total number of counties and the total number of county-days represented in our sample. In Table 2, we report the earliest and the latest ‘first protest’ date within each state’s counties, along with the size of the protest, according to media reports. We provide a map of the continental U.S. in Fig. 1, which reveals the geographic distribution of counties where protests took place along with the size of the first protest that took place within them. Figure 2 shows the evolution of our social distancing restrictions index for a selection of states. Figure 3 shows the social mobility index for a small and a large county in the states of New York and Alabama.

**Table 1:** Sample description

State	New cases		Cumulative cases			Num. counties	County-days
	Mean	Median	Mean	Median	Total		
Alabama	6	13	692	372	46,348	67	8635
Alaska	1	0	46	8	1435	7	864
Arizona	11	4	7006	2048	105,094	15	1900
Arkansas	6	0	315	79	23,598	72	9230
California	3	1	5095	607	295,506	54	6896
Colorado	6	0	541	52	34,647	43	5353
Connecticut	10	8	5851	1414	46,806	8	1032
Delaware	17	16	4105	4977	12,316	3	387
Florida	5	2	3188	646	213,563	67	8622
Georgia	8	3	582	194	92,527	148	18,908
Hawaii	0	0	211	95	1053	3	387
Idaho	2	0	194	30	8538	32	4019
Illinois	4	0	1455	47	148,397	93	11,981
Indiana	5	3	529	162	48,626	92	11,804
Iowa	11	1	325	71	32,137	92	11,745
Kansas	6	0	161	14	16,860	62	7850
Kentucky	2	0	146	39	17,519	112	14,333
Louisiana	11	3	1066	398	68,230	64	8194
Maine	2	0	215	37	3435	16	2064
Maryland	11	9	2933	676	70,396	24	3096
Massachusetts	9	7	7486	7402	104,797	14	1765
Michigan	3	0	837	83	69,463	75	9670
Minnesota	7	0	449	64	39,048	73	9381
Mississippi	11	6	393	282	32,214	77	9890
Missouri	2	0	197	24	22,701	100	12,745
Montana	0	0	24	4	1327	22	2625
Nebraska	6	0	216	9	20,075	45	5629
Nevada	1	0	1399	38	23,785	11	1418
New Hampshire	3	1	593	87	5931	10	1290
New Jersey	13	10	8305	6871	174,407	21	2709
New Mexico	4	0	388	61	12,799	28	3589
New York	5	3	6464	262	400,746	61	7869
North Carolina	7	3	782	362	78,207	97	12,468
North Dakota	2	0	75	6	3973	16	2034
Ohio	4	2	669	130	58,904	88	11,316
Oklahoma	3	0	224	52	17,220	70	8976
Oregon	1	0	295	97	10,605	29	3741
Pennsylvania	3	1	1422	157	95,242	64	8256
Rhode Island	14	7	3101	538	15,503	5	645
South Carolina	5	2	1029	450	47,352	46	5933
South Dakota	10	0	109	14	7163	18	2275
Tennessee	4	0	564	104	53,548	93	11,925
Texas	2	0	832	60	211,326	210	26,696
Utah	3	0	723	0	20,953	23	2924
Vermont	1	0	89	50	1249	12	1548
Virginia	7	0	502	118	66,740	73	9346
Washington	2	0	971	165	37,883	33	4249
West Virginia	2	0	64	22	3505	44	5589
Wisconsin	3	0	452	74	32,556	67	8607
Wyoming	3	0	74	27	1709	18	2295
Total	5	0	941	90	2,957,962	2617	334,703

This table reports the mean and the median number of new COVID-19 cases, per day, per 100,000 population, during the week preceding the onset of the protests (May 18–24, 2020), as well as the mean, median, and total number of confirmed cases, across all counties within each state at the end of our sample period, on July 7, 2020. The number of counties and county-days represented in our sample within each state are reported in the last two columns of the table

**Table 2:** List of U.S. protests

State	First date		Number of participants	
	Earliest	Latest	Smallest	Largest
Alabama	2020-05-29	2020-06-01	50	1000
Alaska	2020-05-30	2020-06-06	20	1400
Arizona	2020-05-28	2020-06-02	50	1000
Arkansas	2020-05-30	2020-06-01	100	1000
California	2020-05-28	2020-06-03	100	3000
Colorado	2020-05-28	2020-06-04	50	1000
Connecticut	2020-05-29	2020-05-31	100	1000
Delaware	2020-05-30	2020-06-01	30	1000
District of Columbia	2020-05-29	2020-05-29	1000	1000
Florida	2020-05-29	2020-06-06	30	1200
Georgia	2020-05-29	2020-06-01	50	1000
Hawaii	2020-05-30	2020-05-30	100	150
Idaho	2020-05-30	2020-06-03	25	1000
Illinois	2020-05-29	2020-06-06	15	1400
Indiana	2020-05-29	2020-06-04	100	10,000
Iowa	2020-05-29	2020-06-05	20	1000
Kansas	2020-05-30	2020-06-06	25	2000
Kentucky	2020-05-28	2020-05-31	100	1000
Louisiana	2020-05-29	2020-06-04	25	1000
Maine	2020-05-29	2020-06-07	100	1000
Maryland	2020-05-29	2020-06-03	100	1000
Massachusetts	2020-05-28	2020-06-02	25	5000
Michigan	2020-05-28	2020-06-01	100	5000
Minnesota	2020-05-26	2020-06-02	100	5000
Mississippi	2020-05-28	2020-05-30	25	1000
Missouri	2020-05-29	2020-06-07	100	2000
Montana	2020-05-29	2020-05-31	50	1000
Nebraska	2020-05-29	2020-06-03	20	5000
Nevada	2020-05-29	2020-06-06	20	1000
New Hampshire	2020-05-30	2020-06-03	100	1000
New Jersey	2020-05-30	2020-06-06	35	10,000
New Mexico	2020-05-28	2020-06-01	40	1000
New York	2020-05-28	2020-06-07	100	11,000
North Carolina	2020-05-29	2020-06-04	25	1000
North Dakota	2020-05-30	2020-06-04	50	1000
Ohio	2020-05-28	2020-06-05	30	5000
Oklahoma	2020-05-30	2020-06-03	1000	1000
Oregon	2020-05-28	2020-06-04	10	2000
Pennsylvania	2020-05-30	2020-06-07	15	5000
Rhode Island	2020-05-30	2020-06-06	100	1000
South Carolina	2020-05-30	2020-05-31	300	1000
South Dakota	2020-05-29	2020-06-05	30	1000
Tennessee	2020-05-27	2020-05-31	50	5000
Texas	2020-05-29	2020-06-06	50	5000
Utah	2020-05-30	2020-05-31	100	1000
Vermont	2020-05-30	2020-06-03	100	1200
Virginia	2020-05-29	2020-06-07	15	1500
Washington	2020-05-29	2020-06-05	100	2000
West Virginia	2020-05-30	2020-05-31	50	1000
Wisconsin	2020-05-29	2020-06-03	100	1000
Wyoming	2020-05-29	2020-06-03	10	1000

This table reports the earliest and the latest date at which the first protest took place in any county within a particular state, as well as the smallest and the largest number of participants reported to have taken part in this first protest

### Regression specification

We examine the impact of the spontaneous relaxation of social distancing behavior that was brought on by the George Floyd protests across the U.S. on the SARS-CoV-2 incidence rate with an Ordinary Least Squares (OLS) staggered differences-in-differences (DID) panel regression equation, which is specified as follows:

1 $$IR_{i,j,t} = \alpha + \beta_{1} Post_{FPi,j,t} + {{\mathbf{X}}_{i,j,t}^{\prime}}\delta_{C1}+{{\mathbf{Y}}_{j,t}^{\prime}}\delta_{C2}+\gamma_{i}+ \eta_{t} +\epsilon_{i,j,t},$$

where $IR_{i,j,t}$, the incidence rate, corresponds to the number of new SARS-CoV-2 infections in county *i* from state *j* on day *t*, per 100,000 population. $Post_{FPi,j,t}$ is an indicator variable that is set equal to one on the day where protests begin in county *i*, as well as every day thereafter, and to zero otherwise. This indicator variable is set to zero on each day *t* for non-protest counties included in our control group. ${\mathbf {X}}_{i,j,t}$ and ${\mathbf {Y}}_{j,t}$ are vectors of county-level and state-level characteristics, which we use as control variables. $\gamma _{i}$ captures time-invariant state fixed effects, and $\eta _{t}$ represents time (day) fixed effects to control for changes in the aggregate SARS-CoV-2 incidence rate and common trends between our treatment and control group counties over time.

In Eq. (1), $\alpha$ is a constant term and $\beta _{1}$ captures the impact of the relaxation of social distancing brought on by the protests on the SARS-CoV-2 incidence rate. Hence, $\beta _{1}$ is the parameter of interest in this regression. Under the null hypothesis that the relaxation of social distancing behavior has no causal impact on the SARS-CoV-2 incidence rate, $\beta _{1}$ should be statistically indistinguishable from zero. We cluster the standard errors at the county level to account for any potential cross-sectional and time-serial dependence in the error terms, $\epsilon _{i,j,t}$ [26, 27]. We perform our statistical analysis with STATA 16 and use and use the REGHDFE command to estimate Eq. (1) [28].

### Covariates

We include county-level control variables that may influence the incidence rate of SARS-CoV-2 in our staggered DID regression specification. These control variables account for demographic, health, living proximity, and income level variations across counties. For demographic controls, we include sex (Male) and age (60 years+) since these factors are associated with both an increased risk of testing positive for SARS-CoV-2 and greater illness severity [29]. We also include ethnicity, i.e., Asian, Black, Hispanic, and White, as demographic control variable, to account for the increased risk of a positive SARS-COV-2 test observed among certain ethnicities, especially Blacks and Hispanics. Our demographic variables are expressed in decimals, and represent the fraction of a county’s total population that falls in a particular group, based on the U.S. Census Bureau’s county-level population statistics for 2018. We include Diabetes prevalence, Hypertension prevalence, Obesity prevalence, and Smoking prevalence as health control variables. Obesity, diabetes, and hypertension are clinical risk factors that are associated with an increased risk of severe illness, and a greater risk of mortality from COVID-19 [30]. Smoking is also a clinical risk factor, as some evidence suggests that smoking may be associated with an increased severity of COVID-19 [31]. We include the natural logarithm of population density, ln(Population density), among our control variables, as higher incidence rates of SARS-CoV-2 are observed in more densely populated, urban, areas [30, 32]. Finally, consistent with previous research showing that residents from more economically deprived areas are more likely to test positive for SARS-COV-2, we use the natural logarighm of real GDP per capita, ln(Per capita RGDP), to control for income in our regressions [30].

In the period preceding the onset of the protests, the number of new COVID-19 cases began to drop steadily across the country [3]. Accordingly, several states began to relax their social distancing restrictions in a carefully staged manner. Figure 2 illustrates this trend in Alabama, California, Florida, and New York, for instance. Starting in mid-March, we observe a steady rise in our social distancing restrictions index in these four states and we observe the start of a slow unwind by mid-April. Notably, while social distancing restrictions were being relaxed across the nation, social mobility was also on the rise (see Fig. 3). The concurrent relaxation of social distancing restrictions and the increase in social mobility around the onset of the protests may very well have contributed to an increase in the SARS-CoV-2 incidence rate during the event period that is unrelated to the protests, so we include our social distancing restrictions and social mobility indexes in our DID regression equation (1), as additional control variables.

### Propensity score matching

The first panel of Table 3 reveals statistically significant differences between protest and non-protest counties included in our sample on just about every dimension represented by the covariates introduced in the previous sub-section, barring the proportion of blacks included in the two groups. Non-protest counties have a significantly higher proportion of males, whites, 60-years+, are less healthy and wealthy, live in less densely populated areas, and are significantly more socially mobile than their counterparts from protest counties. These differences between the two groups may introduce selection bias into our experiment. This is a common concern with observational studies, such as the present one, where the subjects are not randomly assigned to the treatment and control groups by the researcher [33]. To ensure that our control group is as similar as possible to our treatment group from the perspective of all these covariates, i.e., to mimimize any potential selection bias in our experiment, we form our treatment and control groups using the propensity score matching technique [34]. In the context of our experiment, the propensity score represents the estimated likelihood that a county will experience an increase in its SARS-CoV-2 infection rate.

**Table 3:** Summary statistics for covariates

	Unmatched			Propensity score matched		
	Non-protest	Protest	Difference	Non-protest	Protest	Difference
Males	0.501	0.495	0.006***	0.495	0.497	− 0.001
Asian	0.011	0.038	− 0.027***	0.024	0.026	− 0.002
Black	0.099	0.109	− 0.01	0.088	0.096	− 0.008
Hispanic	0.085	0.125	− 0.040***	0.111	0.113	− 0.002
White	0.850	0.819	0.031***	0.850	0.839	0.01
60-years+	0.261	0.233	0.029***	0.242	0.240	0.002
Diabetes prevalence	0.108	0.099	0.010***	0.099	0.101	− 0.002
Hypertension prevalence	0.401	0.377	0.024***	0.378	0.383	− 0.005**
Obesity prevalence	0.389	0.354	0.035***	0.364	0.366	− 0.003
Smoking prevalence	0.245	0.208	0.036***	0.217	0.220	− 0.003
ln(Population density)	3.784	5.378	− 1.593***	4.808	4.763	0.045
ln(Per Capita RGDP)	10.480	10.789	− 0.309***	10.659	10.683	− 0.023
Social distancing restrictions	4.891	5.050	− 0.159*	5.006	4.896	0.11
Social mobility	1.552	0.675	0.877***	0.979	1.024	− 0.045
Number of counties	2077	541		356	356	

This table reports the mean value of the covariates described in “Covariates” section, for non-protest versus protest counties for all the counties included in our sample (unmatched), in the first panel, and for the propensity score matched counties, in the second panel. ***, **, and * denote the statistical significance of *t*-statistics from tests of the null hypothesis that the difference between the means for non-protest and protest counties is statistically indistinguishable from zero at the 1%, 5%, and 10% level, respectively

Essentially, the matching process begins with a logistic regression in which the dependent variable is set to one for the 541 protest (treated) counties included in our sample, and to zero for the remaining 2077 non-protest (untreated) counties. The independent variables included in this regression correspond to our covariates, all of which have been shown to influence the likelihood of contracting SARS-CoV-2. Next, we match treated counties to their nearest neighbour from the untreated group, without replacement, with standard caliper of 0.25 standard deviations, based on the propensity scores from the logistic regression [35, 36]. This process yields a balanced sample consisting of 356 treated and 356 untreated counties. As Table 3 shows, from the perspective of our covariates, these two groups do not exhibit any statistically significant differences from each other, with the exception of Hypertension prevalence, which is significantly higher in our treatment group than in our control group, albeit at the 5% level.

Our quasi-natural experimental setting satisfies at least two key requirements for the identification of the causal link between social distancing and the spread of SARS-CoV-2, namely: (1) the existence of a strong theoretical basis supporting the relationship in question and, (2) exogenous variation in the variable of interest, i.e social distancing [37]. The presence of an exogenous shock in our setting, i.e., protests arising spontaneously in some counties as a result of a tragic event, is key to establish causality, as this mitigates concerns that omitted variables correlated with both the protests and the spread of SARS-CoV-2 might be driving our findings. This setting also minimizes concerns about endogeneity and self-selection, which beset most non-randomized-trial experiments.

In sum, thanks to the covariate balance that we are able to achieve with our propensity score matching process, our staggered DID regression specification is uniquely well positioned to separate the impact of the relaxation of social distancing behaviour on the SARS-CoV-2 incidence rate from other factors that may potentially affect the spread of the disease. Next, to address any potential concerns that our findings may be contaminated by confounding events, we exclude from our regression the county-days that fall outside of the [− 30, + 30]-day event window centered on the day when protests begin in a protest county [33, 38].

## Results

### Impact of protests on SARS-CoV-2 incidence

We report results from regression equation (1) in Table 4. The coefficient of interest in this regression is $\beta _1$, which is associated with $Post_{FP}$, our post-protest indicator variable. This coefficient is positive and highly statistically significant (1.34; 95% CI 0.21–2.47), implying that the SARS-CoV-2 incidence rate increases by 1.34 cases per day, per 100,000, on average, following the onset of the protests in protest counties, relative to their propensity score matching non-protest counterparts. To put this finding into perspective, recall that the average number of new cases across all counties is equal to 5 per day, per 100,000 population, in the week preceding the onset of the protests (see Column (2) of Table 1). Using this number as a reference point, this finding suggests that the SARS-CoV-2 incidence rate increases by 1.34/5 = 26.8% following the onset of the protests, due to the relaxation of social distancing brought on by the protests.

**Table 4:** Impact of protests on SARS-CoV-2 infections

Variables	(1)
$Post_{FP}$	1.34 (0.21–2.47)
Males	59.63 (− 53.94 to 173.20)
Asian	− 38.81 (− 72.57 to − 5.05)
Black	− 25.94 (− 52.91 to 1.04)
Hispanic	21.11 (9.69–32.53)
White	− 32.17 (− 56.31 to − 8.04)
60-years+	5.95 (− 10.17 to 22.08)
Diabetes prevalence	− 58.78 (− 161.35 to 43.78)
Hypertension prevalence	30.15 (− 14.26 to 74.56)
Obesity prevalence	22.79 (1.56–44.01)
Smoking prevalence	− 7.93 (− 48.81 to 32.96)
ln(Population density)	0.80 (0.16–1.45)
ln(Per Capita RGDP)	− 0.02 (− 1.47 to 1.43)
Social distancing restrictions	0.29 (− 0.01 to 0.59)
Social mobility	− 1.20 (− 2.06 to − 0.34)
Constant	− 12.62 (− 77.27 to 52.03)
State fixed effects	Yes
Day fixed effects	Yes
County-days	43,387
Adjusted $R^{2}$	0.10

This table reports results from our staggered DID regression equation (1). In this regressions, the dependent variable corresponds to the county-level number of new confirmed COVID-19 cases, per day, per 100,000 population. $Post_{FP}$ is an indicator variable set equal to zero up until the first protest date in a protest county and to one on every subsequent date. This indicator is set to zero on all dates for the propensity score matching non-protest counties. The 95% confidence intervals reported under the regression coefficients are based on standard errors that are clustered at the county level [26]

Even if our observed covariates are well-balanced, one still needs to assess whether the parallel trends assumption underpinning the DID design is satisfied. We assess whether pre-treatment trends for our treatment and control groups are parallel by estimating a “leads and lags model” [39]. In this model, we replace our $Post_{FP}$ indicator variable in Eq. (1) with a family of period-specific indicator variables spanning the pre- and post-protest event window. Each indicator variable is set equal to one for treated counties for a specific 5-day period surrounding the onset of the protests, and to zero otherwise. Under the null hypothesis that pre-treatment trends are parallel, the coefficients associated with the pre-treatment indicator variables should not exhibit any pattern and should be statistically insignificant. Meanwhile, the coefficients associated with the post-treatment indicator variables will reveal the treatment effect as it manifests itself in the data during the post-protest period.

**Fig. 4:**
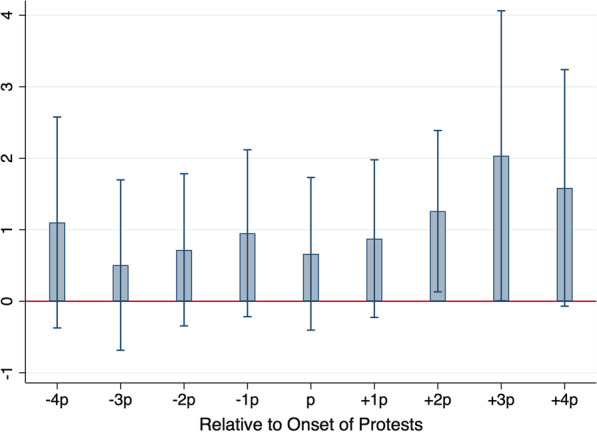
Timing of the protests’ impact on the SARS-CoV-2 incidence rate. Each bar provides the point estimate of the difference between the SARS-CoV-2 incidence rate in protest counties relative to their propensity score matched non-protest counterparts, for 5-day periods around the onset of the protests. For instance, p corresponds to the period starting on the day of the protests and ending four days later, i.e., days [0, 4], + 1p is for day [5, 9], and − 1p is for days [− 5, − 1]. The 95% confidence band is superimposed on each point estimate

Figure 4 plots the value of the coefficients associated with our pre- and post-protest indicator variables. In this figure, p corresponds to the five-day period starting on the protest date and ending 4 days later, i.e., [0, 4], + 1p is for days [5, 9], and -1p is for days [5, 1]. We don’t observe any clear trend in the pre-treatment periods and none of the coefficients are statistically different from zero, suggesting that the parallel trends assumption is satisfied. Post-protest, we observe a clear upward trend in the magnitude of the coefficients, which is reversed in period + 4p. The treatment effect becomes statistically different from zero in period + 2p, roughly ten days following the onset of the protests. This is consistent with SARS-CoV-2’s incubation period and typical testing wait times. Finally, we note the attenuation of the treatment effect in period + 4p. This is to be expected, as the impact of the relaxation of social distancing brought on by the protests must eventually die out. In sum, the treatment effect documented in Table 4 unfolds over time in a manner that supports the hypothesis that social distancing causally impacts the spread of SARS-CoV-2.

### Placebo test

We conduct a placebo test to assess whether the causal impact of the protests on the spread of SARS-CoV-2 that we document in Table 4 can be attributed to chance. For this purpose, we implement a Monte Carlo simulation exercise centered on our staggered DID panel regression specification, i.e., Eq. (1). In each iteration of this simulation, we assign 541 counties randomly to the potential treatment group and the remaining 2077 counties to the potential control group. We then implement our propensity score matching process to create a balanced sample of treated and control counties. We perform this matching process without replacement with the 0.25 standard deviation caliper, as per “Propensity score matching” section. Next, we assign a [− 30, + 30]-day event period to each treated county randomly with start dates ranging between March 1, 2020, and May 8, 2020. Then, we align each control group county’s timeline to its treated counterpart’s event timeline and create the $Post_{FPi,j,t}$ indicator variable. Once this step has been completed, we estimate our staggered DID regression specification on the simulated sample and collect the $\beta _{1}$ coefficient estimate, along with its county-cluster robust *t*-statistic. We implement this process 5000 times to produce the simulated distribution of $\beta _{1}$ coefficients and associated statistics. If the $\beta _{1}$ estimate from Table 4 lies above the 95% threshold from the distribution of simulated $\beta _{1}$ coefficient estimates, we can conclude with a high level of confidence that the treatment effect that we document in this paper cannot be attributed to chance.

We present results from this placebo test in Table 5. The 95% and 99% threshold values for the $\beta _{1}$ coefficient from the simulated distribution are equal to 0.57 and 1.42, respectively, while our empirical estimate in Table 4 is equal to 1.34. Likewise, the 95% and 99% threshold values for the robust *t*-statistics from the simulated distribution are equal to 0.44 and 1.01, respectively, while the robust *t*-statistic associated with our $\beta _{1}$ coefficient estimate in Table 4 is equal to 2.32. Since our $\beta _{1}$ estimate and its associated robust *t*-statistic are well beyond their respective 95% simulated threshold values, we can safely reject the null hypothesis that relaxing social distancing behavior has no impact on the spread of SARS-CoV-2 and, with a high degree of confidence, we can rule out the possibility that the treatment effect that we document in Table 4 is attributable to chance.

**Table 5:** Placebo tests

Coefficient	Mean	Min	p1	p5	p10	p25	p50	p75	p90	p95	p99	Max
Panel A: Random protest onset date and and counties where protests took place												
$Post_{FP}$	− 1.18	− 4.82	− 3.46	− 2.82	− 2.48	− 1.87	− 1.20	− 0.49	0.15	0.57	1.42	2.99
*t*-statistic	− 1.21	− 5.77	− 3.70	− 2.99	− 2.57	− 1.93	− 1.19	− 0.44	0.13	0.44	1.01	2.59
Panel B: Estimates from Table 4												
$Post_{FP}$	1.34											
*t*-statistic	2.32											

This table reports results from a Monte Carlo simulation of the impact of the protests on the SARS-CoV-2 infection rate across the U.S. In each iteration of this simulation, we assign 541 counties randomly to the potential treatment group and the remaining 2077 counties to the potential control group. We then implement our propensity score matching process to create a balanced sample of treated and control counties. Next, we assign a [− 30, + 30]-day event period to each treated county randomly with start dates ranging between March 1, 2020, and May 8, 2020. Then, we create the $Post_{FPi,j,t}$ indicator variable. Finally, we estimate our staggered DID regression specification on the simulated sample and collect the $\beta _{1}$ coefficient estimate, along with its county-cluster robust *t*-statistic [26]. We implement this process 5000 times to produce the simulated distribution of $\beta _{1}$ coefficients and their associated *t*-statistics. We describe this process in greater detail in “Placebo test” section. In Panel A, we report the simulated distribution of the $\beta _{1}$ coefficients, along with the distribution of their *t*-statistics. In Panel B, we report the $\beta _{1}$ estimate from Table 4 to facilitate comparisons

## Discussion

In this paper, we exploit the quasi-natural experimental setting created by the spontaneous relaxation of social distancing brought on by the protests that erupted across the U.S. following George Floyd’s tragic death on May 25, 2020, to the assess the causal impact of social distancing on the spread of SARS-CoV-2 in the U.S. Using a staggered difference-in-difference specification and a balanced sample covering the [− 30, + 30]-day event window centered on the onset of the protests, we document an increase of 1.34 cases per day, per 100,000 population, in the SARS-CoV-2 incidence rate in protest counties, relative to their propensity score matching non-protest counterparts. This represents a 26.8% increase in the incidence rate relative to the week preceding the onset of the protests.

### Strengths and weaknesses

Early predictive models assessing the effectiveness of social distancing have suggested that a greater spread of SARS-CoV-2 would occur in the absence of social distancing measures [40–42]. Similarly, our study demonstrates that when social distancing is reduced, i.e., by individuals protesting in close proximity, the spread of SARS-CoV-2 increases. Our study differs from its predecessors because instead of examining the effectiveness of social distancing measures following their imposition [11, 12, 14], we examine the impact of social distancing on the spread of COVID-19 when social distancing behavior is abruptly relaxed. Additionally, unlike previous studies, we do not use mobility as a measure of social distancing, instead we use social mobility as a control variable in our analyses. By explicitly controlling for the concurrent increase in social mobility and the relaxation of state-imposed social distancing restrictions during the period surrounding the protests, our study demonstrates that social distancing directly impacts the spread of SARS-CoV-2. We also control for a host of covariates known to influence the transmission of SARS-CoV-2, and implement placebo tests to rule out the possibility that our results are attributable to chance. Therefore, we can be confident that the increase in SARS-CoV-2 incidence that we observe following the onset of the protests can be attributed to the relaxation of social distancing behavior.

Our study is not without limitations. In particular, over 70 testing centers across the U.S. were closed following the onset of the protests. We are also unable to assess protest participants’ vulnerability (e.g. age, underlying health conditions, personal protective wear, etc.), and variability along these dimensions may influence the risk of SARS-CoV-2 incidence. Additionally, we cannot control for the actual degree of physical proximity between participants, which would impact the transmission rate of SARS-CoV-2 during the protests. We are also unable to control for any potential under-reporting of COVID-19 cases over time and across counties [43]. This would be a concern if protest counties and non-protest counties were impacted differently by this phenomenon. Moreover, we rely on the accuracy of media reports to identify the counties in which protests took place. Finally, we do not account for the magnitude of the protests in each county, however, expressing the case counts in rates rather than in levels should minimize any potential scale-related effects.

#### Future research and implications

Future research using this experimental setting could use machine learning tools to analyze protest videos and determine the relative contribution of participant demographics, the degree of physical distancing, and the extent and type of personal protective wear on the spread of SARS-CoV-2. Social mobility data might also be used to track the extent to which people who participated in protests visited a SARS-CoV-2 testing centres at any point before or after they partook in protests. Taken together, this study demonstrates that, when controlling for social mobility restrictions, social mobility, and a host of other potential risk factors for the contraction of SARS-CoV-2, the relaxation of social distancing behavior causally impacts the spread of SARS-CoV-2. As states are in the midst of relaxing the social distancing restrictions initially imposed in March 2020, establishing the effectiveness of social distancing behavior in a statistically reliable way has important public health implications. Our research informs policy makers and provides insights regarding the usefulness of social distancing as an intervention to minimize the spread of SARS-CoV-2, and reduce the risk of a second, and possibly, third wave of COVID-19.

## Data Availability

All the studies cited in this paper are peer-reviewed journal articles or preprints and can be accessed in the public domain. All datasets utilized to conduct this experiment (U.S. covid-19 data, county-level demographic data, social distancing restrictions, and the list of protests data) are accessible publicly and links to these sources are provided in the list of references. The Stata dataset that was constructed for this study is available from the authors upon request.
